# Perceived value and tourist donation intentions at religious heritage sites: the mediating roles of awe and subjective well-being

**DOI:** 10.3389/fpsyg.2025.1655120

**Published:** 2025-09-12

**Authors:** Shuo Yu, Xi Li, Yu Cao

**Affiliations:** ^1^Faculty of International Tourism and Management, City University of Macau, Taipa, Macao SAR, China; ^2^Faculty of Hospitality and Tourism Management, Macau University of Science and Technology, Taipa, Macao SAR, China

**Keywords:** religious heritage tourism, perceived value, donation intention, awe, subjective well-being, religiosity

## Abstract

**Introduction:**

Religious heritage tourism is increasingly recognized for its dual contribution to cultural preservation and economic sustainability. However, the psychological mechanisms that drive tourists' willingness to donate at religious heritage sites remain underexplored. Building on the Stimulus-Organism-Response (SOR) theory, this study investigates how tourists' perceived value shapes their donation intentions, highlighting the mediating roles of awe and subjective wellbeing, as well as the moderating role of religiosity.

**Methods:**

Data were collected from 529 visitors to the A-Ma Temple in Macau. Partial Least Squares Structural Equation Modeling (PLS-SEM) was applied to test the hypothesized relationships among perceived value, awe, subjective wellbeing, religiosity, and donation intention.

**Results:**

The findings reveal that perceived value has a positive influence on donation intentions, both directly and indirectly, through awe and subjective wellbeing. Furthermore, religiosity significantly moderates these relationships, such that individuals with higher religiosity exhibit stronger effects of awe and subjective wellbeing on donation intentions.

**Discussion:**

By incorporating the SOR framework, this study advances theoretical understanding of the psychological mechanisms underlying donation behavior in religious heritage tourism. The results suggest that enhancing tourists' emotional and spiritual experiences can strengthen their willingness to provide financial support, offering practical insights for heritage site managers seeking sustainable conservation strategies.

## 1 Introduction

As an important component of global tourism, religious tourism represents a distinctive travel form that is fundamentally intertwined with faith and cultural traditions (Baek et al., 2022). It also demonstrates unique values in economic, social, and cultural complexity (Romanelli et al., 2021). Religious tourism forms and functions are evolving to cater for the diverse tourist demands in the contemporary world, which embraces both conventional pilgrimages and modern experiences that emphasize heritage conservation, cultural interaction, and spiritual fulfillment (Wang et al., 2010; Xu and Hu, 2024). This transition highlights the adaptability and multifunctionality of religious tourism in modern society. The process of traveling for religious purposes has transformed sacred heritage into a primary resource for tourism, attracting visitors through profound historical significance, cultural symbolism, and spiritual meaning. Major pilgrimage destinations such as Jerusalem and Mecca embody the core values of Judaism, Christianity, and Islam, and have developed large industries that provide services for pilgrims and other visitors (Dan, 2011). These sites not only attract millions of visitors but also drive economic growth and foster cultural dialogue among host communities and tourists (UNWTO, 2014). In China, there is a growing interest in religious heritage tourism. Tourists' interest in religious heritage has steadily grown through the restoration and protection of religious cultural sites, bringing significant economic benefits and social impacts to the destinations (Zhang and He, 2025). Particularly, temple sites are increasingly attractive as they offer rich opportunities for historical and cultural interactions at the destination (Kastenholz and Gronau, 2022). One such site is the A-Ma Temple in Macau, the oldest temple in the region, built in 1488 (Veronica, 2025). As a UNESCO World Heritage site, A-Ma Temple integrates Taoist, Buddhist, and folk beliefs, making it an exemplary location for investigating tourists' perceptions and behaviors in religious heritage tourism (Veronica, 2025).

In recent years, research on religious tourism and religious heritage has made great progress. Nevertheless, notable gaps remain. Existing research primarily focuses on the conservation and management of religious heritage sites (Mekonnen et al., 2022; Sabri and Olagoke, 2019), their evolution and development (Iliev, 2020; Kim et al., 2020), as well as their economic impacts (Budovich, 2023; Zhang and He, 2025). In contrast, investigations into tourists' emotional experiences, psychological mechanisms, and behavioral intentions are relatively scarce. For example, few studies have examined awe in religious heritage tourism, despite its role as a key part of tourists' spiritual fulfillment and behavioral transformation (Zhang and He, 2025). Furthermore, few studies have explored the complex network of mechanisms (including awe and subjective well-being) through which perceived value influences the intention to donate at religious sites (Ariza-Montes et al., 2017). Limited research has been conducted on the moderating effect of religiosity on the relationship between tourists' emotional experiences and behavioral intention (Abror et al., 2019). This is especially applicable to some instances of religious heritage tourism in Asia, where further research should be conducted to determine how different cultures interact with religiosity to influence tourist behavior (Chao and Yang, 2018). To address this research gap, the present study intends to develop a more comprehensive theoretical framework for religious heritage tourism and use the framework to analyze the relationship among tourists' perceptions, emotional experiences, and behavioral intentions.

The Stimulus–Organism–Response (SOR) model, rooted in environmental psychology, offers a useful framework for investigating these processes. The SOR paradigm posits that environmental stimuli (S) influence an individual's internal cognitive and affective state (O), which in turn shapes behavioral responses (R) (Pan et al., 2024). Unlike cognitive theories, such as the Theory of Planned Behavior or the Norm Activation Model, which focus on rational intentions and attitudes (Meng et al., 2020; Shin et al., 2018), the SOR model incorporates emotional and sensory reactions. This broader perspective makes it particularly suitable for uncovering the pathways through which tourists' perceived value influences their donation intentions in religious contexts. Owing to its flexibility, the SOR model has been widely applied across diverse fields, including education, marketing, and tourism. In tourism research, the model is frequently used to examine the interaction between stimuli (such as perceived value, Zhao and Weng, 2024), psychological states (e.g., awe and subjective well-being, Xu and Hu, 2024), and behavioral responses (e.g., donation intentions, Li et al., 2024). Religiosity can act as a moderating factor, explaining variations in emotional and behavioral reactions among tourists (Lindridge, 2005; Mukhtar and Butt, 2012). The SOR model's focus on emotions makes it especially useful in studying the emotional and behavioral dynamics of tourists, particularly in religious heritage tourism.

To address the aforementioned research gaps, the present study constructs a comprehensive theoretical framework based on the SOR model. It investigates how tourists' perceived value of A-Ma Temple influences their emotions (awe and subjective well-being) and, in turn, shapes their behavioral intentions (e.g., donation). Specifically, this study explores two questions: (1) How does perceived value shape tourists' donation intentions in the context of religious heritage tourism, with awe and subjective well-being acting as mediators? (2) To what extent does religiosity moderate the relationship between psychological experiences and behavioral intentions? By elucidating the psychological mechanisms through which religious heritage influences tourist behavior, the study aims to provide both theoretical insights and practical recommendations for heritage site managers seeking to balance cultural preservation with visitor expectations.

## 2 Literature review

### 2.1 Theoretical underpinning

The Stimulus-Organism-Response (SOR) model (Mehrabian and Russell, 1974) posits that external stimuli (S) influence internal psychological states (O), which in turn shape behavioral responses (R). This framework emphasizes the mediating role of internal states and has been widely applied in tourism research, including studies on support for religious tourism (Hung Lee et al., 2021), virtual tourism intentions (Jiang et al., 2024), tourism shopping intentions (Hew et al., 2018), and augmented reality tourism intentions (Zhu et al., 2024). However, most of these studies focus on general behavioral intentions (e.g., revisit or purchase intentions) rather than prosocial behaviors, underscoring the need to explore donation intentions within religious tourism.

In selecting SOR variables, this study adopts perceived value as the stimulus because it integrates visitors' appraisals of both tangible (e.g., infrastructure quality) and intangible (e.g., emotional and social) attributes of religious heritage sites (Yu et al., 2023). As a multifaceted construct, perceived value offers a robust theoretical link between environmental stimuli and subsequent emotional and behavioral responses within the SOR framework, making it a well-established antecedent of tourist emotions and behaviors (Kim and Thapa, 2018). Awe and subjective well-being, as organismic variables, embody complementary emotional and cognitive processes elicited by profound spiritual experiences. Awe captures the transcendental, self-transcending emotion provoked by vast or sacred stimuli (Stamatopoulou et al., 2024), while subjective well-being reflects the cognitive appraisal of life satisfaction and meaning derived from these experiences (Zhao and Weng, 2024). Therefore, these structures accurately reflect the unique psychological processes of tourists in religious tourism. Finally, donation intention is chosen as the behavioral response because donation behavior is a normative and observable act in religious heritage tourism, visitors often make donations to support temple upkeep, cultural heritage preservation, or community welfare (Shinde, 2011). This distinguishes it from secular revisit intentions or word-of-mouth behaviors. Beyond its prevalence, donations create an immediate, site-specific resource flow that sustains daily operations and ritual services and directly finances tangible and intangible heritage conservation (Zhao et al., 2025). Accordingly, modeling donation intention provides destination managers with feasible strategies for the sustainable development of sacred sites, making it a more policy-relevant response variable than post-visit loyalty outcomes alone.

In the context of religious tourism, Yu et al. (2023) identified specific dimensions of perceived value and demonstrated its positive impact on visitor satisfaction. Previous studies have also highlighted a strong linkage between perceived value, awe, and behavioral intentions (Argan et al., 2025; Wang et al., 2021). On this basis, Zhao and Weng (2024) demonstrated that perceived value enhances subjective well-being, which subsequently shapes tourists' behavioral intentions. Similarly, Xu and Hu (2024) found that both emotional responses (e.g., awe) and cognitive reactions (e.g., subjective well-being) experienced in religious sites positively influence tourist behavior. Aji and Muslichah (2023) also confirmed the relevance of donation behavior within religious contexts. Collectively, these findings delineate a clear pathway: from perceived value to awe and subjective well-being, eventually culminating in behavioral intentions. The Stimulus-Organism-Response (SOR) model provides a comprehensive theoretical framework to interpret this process, systematically linking environmental stimuli (i.e., perceived value) with internal psychological mechanisms (i.e., awe and subjective well-being), and ultimately leading to specific behavioral responses (such as donation intentions) within the context of religious heritage tourism.

### 2.2 Religious tourism

Research on religious tourism first emerged in the 1980s (Nolan and Nolan, 1992; Rinschede, 1992). Scholars defined religious tourism as a distinct type of tourism motivated partly or wholly by religious reasons (Nolan and Nolan, 1992; Rinschede, 1992). It involves visiting pilgrimage sites to fulfill both religious and recreational needs (Shinde, 2007). Activities in this segment include missionary trips, monastery visits, and faith camps (Wight and Victoria, 2022), and it attracts both repeat visitors and pilgrims (Terzidou, 2008). Existing studies primarily focus on religious tourist experiences (Bond et al., 2015; Xu and Hu, 2024; Yu et al., 2023), religious tourism motivations (Amaro et al., 2018; Kruger and Saayman, 2016; Wang et al., 2016), and religious beliefs (Abdou et al., 2024; Memon et al., 2020; Royanow et al., 2024). However, few studies explicitly link religious tourism with donation behaviors, leaving a gap in understanding how emotional and spiritual experiences translate into prosocial behaviors.

Recent research emphasizes that religious tourism is not merely a physical journey but involves profound psychological transformation. Pilgrims seek transcendence, connectedness, and inner harmony, which influence their emotional states and well-being (Zhang et al., 2023). Empirical studies in Buddhist contexts show that spiritual experiences foster a deeper sense of meaning and shape future behaviors (Zhang et al., 2023). Awe, experienced when environmental and religious stimuli exceed cognitive expectations, can reconstruct visitors' relationships with the destination and encourage responsible behavior (Qi et al., 2018). In sacred sites, awe enhances cultural attachment and subjective well-being, leading to loyalty (Xu and Hu, 2024). These findings suggest that awe and subjective well-being are key mediators between perceived value and behavioral responses. Moreover, religiosity and spirituality have been identified as positive predictors of subjective well-being (Villani et al., 2019), and religiosity enhances the link between donation intentions and actual donations (Susanto et al., 2021). Despite these insights, limited research systematically examines how perceived value, awe, subjective well-being, and religiosity jointly shape donation intentions in religious tourism. This study addresses this gap by elucidating the psychological mechanisms underlying donation behavior in religious settings.

### 2.3 Tourists' perceived value

Perceived value is widely defined as the overall evaluation of a product's utility based on the trade-off between what is received and what is given (Zeithaml, 1988). In tourism contexts, perceived value encompasses not only functional benefits but also the emotional and psychological benefits generated through interactions between tourists and their environment (Zhang et al., 2023). Tourism scholars have adapted this concept to heritage settings, noting that the perceived value of religious sites includes visitors' assessments of service quality, emotional response, and spiritual significance (Eid, 2015; Eid and El-Gohary, 2015). Unlike tangible goods, tourism experiences possess intangible, diachronic, and interactive characteristics, leading visitors to evaluate value from a holistic perspective (Zhang et al., 2023). Both functional attributes (e.g., infrastructure, cleanliness) and emotional or symbolic attributes (e.g., sacred atmosphere, cultural richness) jointly shape tourists' perceptions. Empirical studies show that perceived value significantly influences preferences, satisfaction, and loyalty (Chen and Chen, 2010) and is determined by both objective attributes and subjective elements such as visitors' motivations, attitudes, and prior experiences (Zhang et al., 2023). These findings justify our selection of perceived value as the external stimulus in the SOR framework: it synthesizes the multiple dimensions of a visitor's appraisal, providing a theoretically grounded antecedent for emotional and behavioral responses.

Perceived value does not merely influence cognitive evaluations; it also elicits affective reactions. High perceived value can evoke positive emotions such as awe when the environment exceeds visitors' cognitive expectations (Wang and Lyu, 2019). Awe often emerges in sacred religious sites, where the solemn atmosphere, sacred architecture and ritual practices trigger feelings of reverence (Qi et al., 2018; Lu et al., 2017). Such emotions enrich tourists' perception of culture and spirituality, transforming a visit into a meaningful, self-transcending experience. For instance, Pearce et al. (2017) observed that vast geological landscapes enhance visitors' awe, and similar effects occur when visitors perceive high quality, emotional and symbolic attributes in religious tourism. Based on that, we propose the following hypothesis:

**H1: Tourists' perceived value positively influences their sense of awe**.

Moreover, some studies emphasize that participation in meaningful activities significantly enhances subjective well-being (Sheldon and Lyubomirsky, 2004). For example, (Lv and Xie 2017) demonstrated that the perceived value of rural tourism among residents directly boosts their subjective well-being, which subsequently enhances their attachment to these places. Similarly, Zhao and Weng (2024) revealed that tourists' perceived value of urban forest parks, spanning aspects like resource quality, service quality, emotional value, and tourism costs, positively influences their subjective well-being. Based on these studies, we propose the following hypothesis:

**H2: Tourists' perceived value positively influences their subjective well-being**.

Several existing studies have demonstrated that tourists' perceived value plays a critical role in predicting consumer satisfaction and behavioral intentions (Chen and Chen, 2010; Eid, 2015; PandŽa Bajs, 2015). Notably, Jang et al. (2025) developed a theoretical model to examine food donation behavior and identified a positive correlation between tourists' perceived value and their intention to donate food. Given these insights, we hypothesize the following:

**H3: Tourists' perceived value positively influences their donation intention**.

### 2.4 Awe and subjective well-being

Awe is a complex positive emotion that occurs when individuals encounter stimuli that surpass their cognitive frameworks (Keltner and Haidt, 2003). It is characterized by perceptions of vastness and the need for cognitive accommodation (Shiota et al., 2007). In the context of religious heritage tourism, awe is evoked not only by natural grandeur but also by sacred architecture, ritual practices, and spiritual symbolism, all of which induce a unique sense of smallness and reverence (Xu and Hu, 2024). This profound emotional experience shifts the focus away from the self, helping to alleviate stress and enhance satisfaction (Bai et al., 2021).

Subjective well-being encompasses both individuals' emotional responses and their cognitive evaluations of life satisfaction and a sense of accomplishment (Armbrecht and Andersson, 2020). Existing research indicates that participation in social activities such as religious festivals and cultural events positively influences subjective well-being (Brownett, 2018; Yolal et al., 2016). Specifically, Filep and Deery (2010) highlight that subjective well-being for tourists is manifested through positive emotions, a sense of involvement, and the meaningfulness they derive from travel experiences.

Awe has been recognized as a key factor contributing to psychological well-being (Bethelmy and Corraliza, 2019). For instance, Liu et al. (2023) reported that awe in mountainous environments significantly improved tourists' well-being, particularly at higher altitudes. Similarly, Xu and Hu (2024) demonstrated that awe experienced at cultural heritage sites significantly enhances tourists' subjective well-being. These findings suggest a close connection between awe and subjective well-being in the tourism context. Building on these insights, we propose the following hypothesis:

**H4: Tourists' sense of awe positively influences their subjective well-being**.

### 2.5 Donation intention

Donation intention refers to the degree to which potential donors are willing to contribute to individuals, communities, or organizations (Hou et al., 2021). The motivations underlying donation behavior are generally categorized into intrinsic and extrinsic factors, encompassing both self-interest and concern for others (Konrath and Handy, 2018; Ye et al., 2015). From a self-interest perspective, individuals' actions, including donations, are often driven by personal desires and the potential for personal gain (Ye et al., 2015). Conversely, altruistic individuals are more likely to convert their personal benefits into acts of assistance for others, thereby promoting social welfare (Schefczyk and Peacock, 2010). Current research on donation intention primarily focuses on charitable donations (Ye et al., 2015), food donations (Jang et al., 2025), and online donations (Hou et al., 2021). In this study, donation intention specifically refers to tourists' willingness to contribute resources (financial or in-kind) to the host temple or heritage organization, with the donated resources being used for the preservation and sustainable management of religious heritage.

Religious temples evoke awe and alter perceptions, motivating pilgrims to act accordingly (Qi et al., 2018). Merit-making rituals often involve donations, such as contributions to monks or offerings for health and prosperity (Shmushko, 2023). According to Fredrickson's broaden-and-build theory (2001), positive emotions like awe expand individuals' thought–action repertoires and increase prosocial behaviors. Subjective well-being, as a positive emotional experience, also serves as a key motivator for donation behavior (Bagozzi et al., 2001). Tourists experiencing higher subjective well-being during religious tourism may be more inclined to contribute to public welfare or heritage preservation efforts (Oz, 2019). Therefore, we propose the following hypothesis:

**H5: Tourists' sense of awe positively influences their donation intention**.

**H6: Tourists' subjective well-being positively influences their donation intention**.

### 2.6 The mediating role of awe and subjective well-being

Building on H1-H3 and H5-H6, we argue that awe and subjective well-being mediate the relationship between perceived value and donation intention. Specifically, awe has been considered an important factor that influences tourists' perceived value (Zhang and He, 2025). Awe increases both emotional responses and behavioral intentions (Qi et al., 2018), and subjective well-being mediates the link between perceived value and behavioral intentions (Zhao and Weng, 2024). These mediators explain how external stimuli translate into prosocial outcomes. Therefore, we propose:

**H7: Awe mediates the relationship between tourists' perceived value and their donation intention**.

**H8: Subjective well-being mediates the relationship between tourists' perceived value and their donation intention**.

### 2.7 The moderating role of religiosity

Religiosity is considered a source of meaning, providing positive emotions that promote mental health and overall happiness (Singh, 2014). Existing studies show that religiosity influences consumer behavior and preferences (Hill et al., 2000; Zinnbauer et al., 1999) and shapes tourists' tastes, perspectives, and actions (Abu-Alhaija et al., 2018; Schänzel and Yeoman, 2015). As an example, attitudes toward purchasing halal items correlate positively with religiosity (Mukhtar and Butt, 2012). Religiosity also affects how people see the world and make life plans (Lindridge, 2005). Higher religiosity correlates with greater happiness and life satisfaction (Lim and Putnam, 2010). Patrick and Kinney (2003) found that religiosity enhances well-being, corroborating its role as a source of positive emotions.

Religiosity functions not merely as a belief system but also as a psychological mechanism that translates cognitive and affective states into prosocial behavior (Van Cappellen et al., 2016). Empirical evidence demonstrates that religiosity positively influences individuals' attitudes and behavioral outcomes (Kamalul Ariffin et al., 2016), particularly in fostering ethical conduct such as pro-environmental actions (Bhuian et al., 2018). Furthermore, religiosity has been shown to moderate the effects of personal and social norms on behavior (Ullah et al., 2024), to strengthen the links between emotions and life satisfaction (Joshanloo, 2019), and to reinforce the relationship between charitable attitudes and donation motives (Teah et al., 2014). In religious tourism, awe, often interpreted as transcendence, heightens moral concern and activates prosocial norms (Piff et al., 2015), while elevated subjective well-being promotes engagement in public-good or heritage-preservation activities (Oz, 2019). When destinations bear sacred meaning, stewardship motivations and willingness to sacrifice are intensified (Tarakeshwar et al., 2001). Taken together, these findings indicate that religiosity can shape value orientations and channels positive emotions into prosocial behavior. Therefore, we posit that high religiosity amplifies the emotional resonance of awe and well-being, making it more likely for these experiences to translate into donation behaviors.

Based on these mechanisms, we hypothesize that religiosity moderates the relationships between awe, subjective well-being, and donation intention:

**H9: Religiosity positively moderates the effect of awe on tourists' donation intention, such that the effect is stronger for highly religious tourists**.

**H10: Religiosity positively moderates the effect of subjective well-being on tourists' donation intention, such that the effect is stronger for highly religious tourists**.

**Figure 1:**
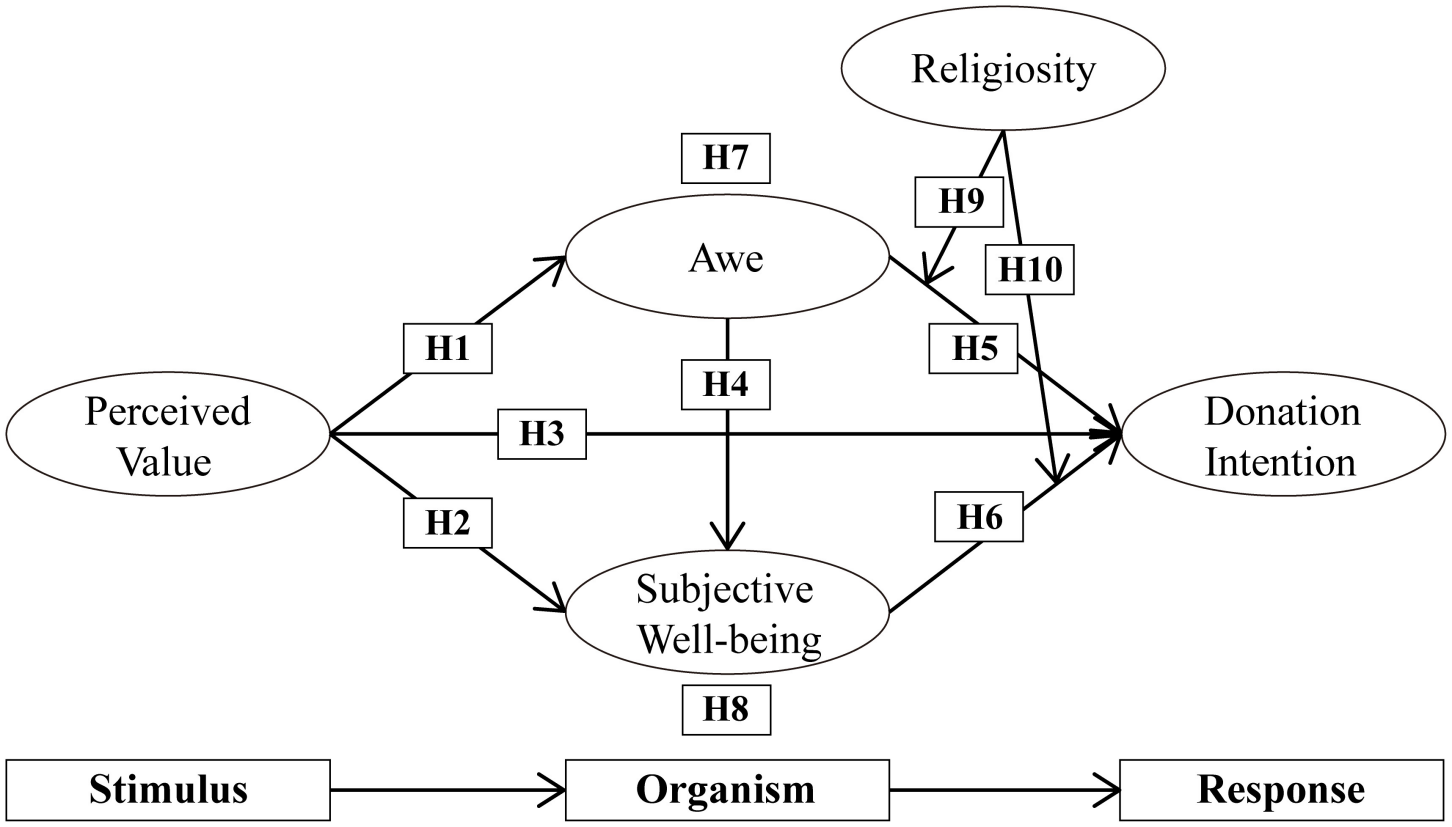
Research framework.

Collectively, these hypotheses build a comprehensive framework (Figure 1) linking perceived value, awe, subjective well-being, and religiosity to donation intention within religious tourism.

## 3 Methods

### 3.1 Research site

**Figure 2:**
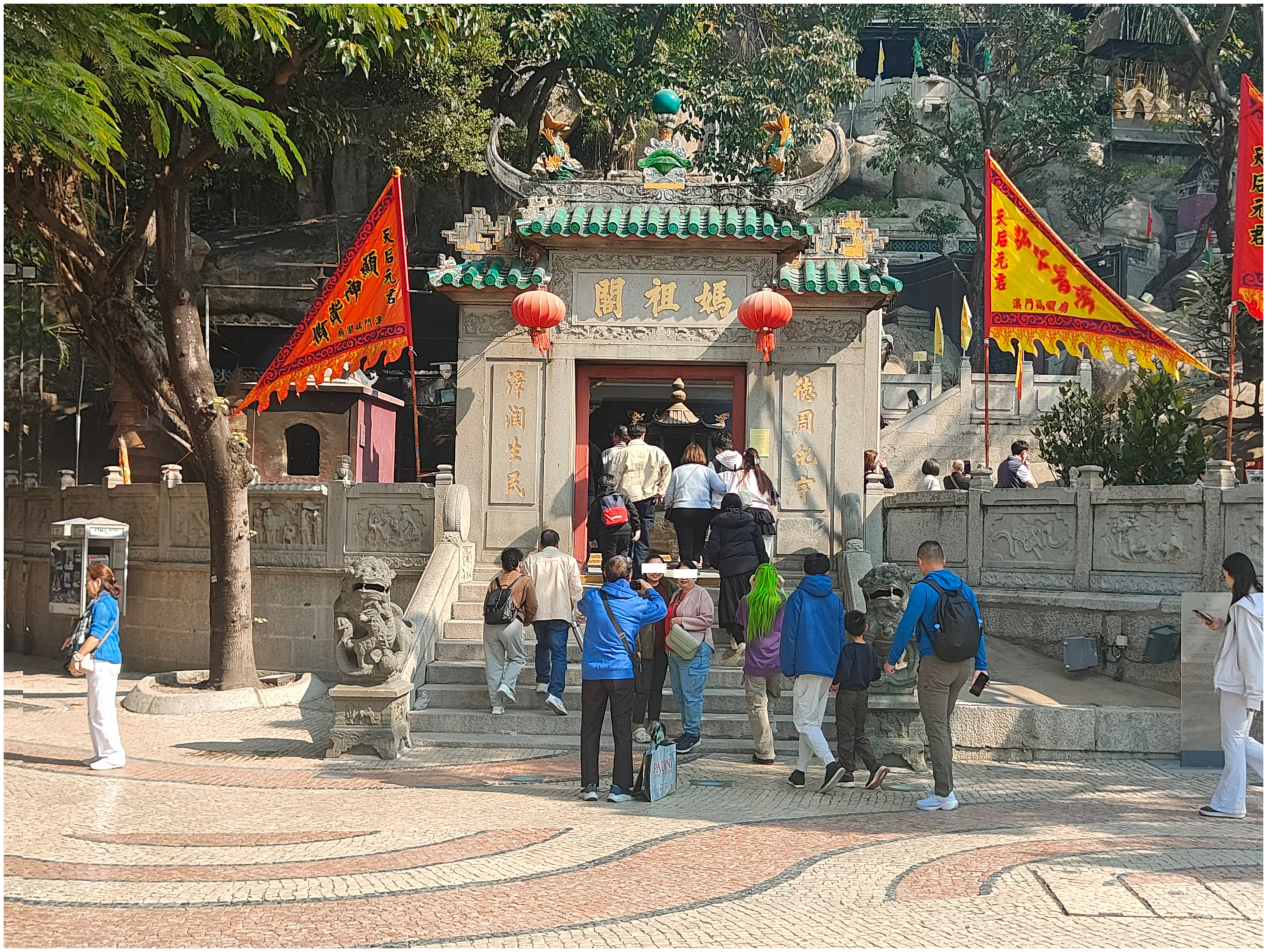
Research site—A-Ma Temple (Photographed by the researcher).

Macau is located in the southern part of the Pearl River Delta and is a Special Administrative Region of China. With over 400 years of historical accumulation, Macau has developed a diverse, inclusive, and integrated religious landscape (Ma and Chen, 2023). In 2005, the Historic Center of Macau was inscribed on the UNESCO World Heritage List (UNESCO, 2005). The Historic Center of Macau centers on the old city, encompassing more than 20 historical buildings, the majority of which are related to religious activities and architecture. The temple architecture in Macau is diverse, including traditional Chinese Taoist and Buddhist temples, as well as distinctive religious buildings that integrate both Chinese and Western architectural styles (Yin and Jia, 2024). Among these, the A-Ma Temple stands out as the most representative example. The A-Ma Temple is one of the oldest and best-preserved temples in Macau. It has undergone centuries of historical transformation, becoming a significant testament to the fusion of religion and culture in Macau (Macao Government Tourism Office, 2021). This uniqueness positions the A-Ma Temple as an ideal location for studying religious heritage tourism (see Figure 2).

### 3.2 Questionnaire design and measurement

The questionnaire consists of three sections. The first section contains a screening question: “Have you visited the A-Ma Temple in the past 6 months?” Only respondents who answer “Yes” are eligible to proceed to the next part of the survey. The second section includes all the structured measurement items. To ensure reliability and validity, all the measurement items are adapted from existing mature scales and were appropriately modified for the context of this study (see Supplementary Table S2). Perceived value was assessed using items adapted from Yu et al. (2023). To align with the sacred context, references to “resort” were reworded as “temple.” For instance, the original item “The environment of the resort was good” was modified to “The environment of the temple was good.” Similarly, “The resort has well-developed infrastructure” was revised to “The temple had well-developed infrastructure,” emphasizing interactions appropriate to religious settings. Items for awe were adapted from Yan and Jia (2021). To ensure relevance to the temple experience, items were rephrased; for example, the bipolar pair “Boring–excited” was framed as “In the temple, I was bored–excited.” Likewise, “Usual–unusual” was modified to “In the temple, I felt usual–unusual.” This wording ensures that the emotion was triggered by the sacred environment, not travel in general. The subjective well-being scale, adapted from Xu and Hu (2024), was used to measure emotional fulfillment during temple visits. As the original items already captured subjective well-being in religious contexts, the scale was retained largely unchanged (e.g., “I feel like my travel is satisfactory and festive” was changed to “I felt like my travel was satisfactory and festive”). Religiosity items from Preston and Shin (2017) were retained with minimal modifications, as they already captured personal religious importance and identity, with slight wording adjustments for consistency (e.g., replacing “religious beliefs” with “religiosity”). Donation-intention items were adapted from Jang et al. (2025). Since the original scale focused on food donation intentions, all items were rephrased to reflect monetary or in-kind donations to the temple. For example, “I intend to donate food in the future” was changed to “I intend to donate resources (financial or in-kind) to the temple in the future,” and “I am willing to contribute by donating food” was revised to “I am willing to contribute by donating resources (financial or in-kind) to the temple.” Awe was assessed using a seven-point semantic differential scale, while the other items were measured using a seven-point Likert scale (1 = strongly disagree, 7 = strongly agree). The third section collects participants' demographic information. The questionnaire underwent a rigorous translation and back-translation process to ensure the accuracy of the translations (Thammaiah et al., 2016). A bilingual questionnaire was first validated by two professors in the field of tourism studies. Then, prior to the official distribution of the questionnaire, a pre-test was conducted with 74 tourists who had visited the A-Ma Temple in the past 6 months. They reported no issues with the content of the questionnaire.

### 3.3 Data collection

A convenience sampling method was utilized at the main entrance of the A-Ma Temple, where two trained research assistants approached and invited tourists who had just completed their visit to participate in the survey. This approach was selected due to the large and diverse flow of tourists at this religious heritage site, thereby ensuring that only individuals with direct, recent, and relevant experiences were included in the sample. Data collection was conducted from November 22 to December 13, 2024. All participants were assured of the anonymity and confidentiality of their responses and were informed that participation was entirely voluntary; they could withdraw from the study at any time without consequence. Of the 565 individuals who completed the questionnaire, 35 were excluded because their responses did not meet the inclusion criteria. As a result, a total of 529 valid questionnaires were retained for data analysis, yielding a response rate of 93.63%.

### 3.4 Data analysis

Quantitative research is well established for its scientific rigor and effectiveness in hypothesis testing (Nardi, 2018). Accordingly, this study adopts a quantitative approach to validate the proposed mechanism through which perceived value influences donation intentions among religious tourists. Partial Least Squares Structural Equation Modeling (PLS-SEM) was employed due to its suitability for examining complex models with multiple latent constructs and interrelationships (Hair Jr et al., 2022). Smart-PLS 4 was selected for its proven capability in handling small sample sizes and supporting exploratory research (Tang et al., 2020). The analysis followed a two-stage procedure: first, the measurement model was evaluated in terms of reliability (Cronbach's α, Composite Reliability), convergent validity (Average Variance Extracted), and discriminant validity (Fornell-Larcker criterion, Heterotrait-Monotrait ratio). Second, the structural model was assessed using a 5,000-sample bootstrap to estimate path coefficients and indirect effects.

## 4 Results

### 4.1 Simple profile

Table 1 presents the respondents' profiles. There were 52% females and 48% males. The age group 18–25 accounted for 24.8%, while the 26–35 age group made up 22.7%. Most respondents had a bachelor's degree, accounting for 57.1%, and 56.7% had a monthly income between 3,001 and 10,000 yuan. Corporate employees made up 36.9% of the respondents.

**Table 1:** Respondent demographics (*N* = 529).

**Variable**	**Category**	**Frequency (*N* = 529)**	**Percent% (*N* = 529)**
Gender	Male	254	48.0
	Female	275	52.0
Age	18–25 years old	131	24.8
	26–35 years old	120	22.7
	36–45 years old	109	20.6
	46–55 years old	92	17.4
	56 years old and above	77	14.6
Education	Junior high school and below	58	11.0
	High School/Secondary School	113	21.4
	University/Junior college	302	57.1
	Postgraduate degree	56	10.6
Income (RMB)	Under 3,000	144	27.2
	3,001–5,000	152	28.7
	5,001–10,000	148	28.0
	Above 10,001	85	16.1
Career	Student	28	5.3
	Company employees	195	36.9
	Self-employed	93	17.6
	Institutions/civil servants	105	19.8
	Service industry personnel	47	8.9
	Workers	17	3.2
	Others	44	8.3

### 4.2 Common method bias

Common Method Bias (CMB) can lead to inflated correlations between variables measured using the same method (Williams and Brown, 1994). Adopting Harman's Single-Factor Test, the results from the unrotated solution showed that the first component explained 33.98% of the variation (lower than the 50% cutoff) (Podsakoff et al., 2012), indicating no evidence of common method bias in this study.

### 4.3 Reliability and validity test

The outcomes of the tests for convergent validity and reliability are presented in Table 2 below. According to Hair et al. (2019), the constructs demonstrate satisfactory reliability and convergent validity, as both the Cronbach's α and Composite Reliability (CR) are higher than 0.7, and the Average Variance Extracted (AVE) is greater than 0.5. According to Table 3, the results of discriminant validity testing yielded valid and reliable outcomes. First, the square root of each construct's AVE is greater than the correlations between it and the other constructs, in accordance with the Fornell-Larcker criterion. Second, all Heterotrait-Monotrait (HTMT) ratios are below 0.85, suggesting adequate discriminant validity (Hair Jr et al., 2022).

**Table 2:** Reliability and validity analysis of scale.

**Construct**	**Item**	**Factor loading**	**CA**	**CR**	**AVE**
Perceived Value (PV)	The environment of the temple was good.	0.748	0.961	0.964	0.561
	The temple had well-developed infrastructure.	0.734			
	The temple staff patiently answered my questions.	0.759			
	The price of the city where the temple locates was reasonable	0.762			
	Transportation to the temple was convenient and affordable.	0.769			
	Spending at temples (meals, cultural creations, etc.) was reasonable.	0.802			
	I was comfortable with this religious tourism.	0.722			
	I felt relaxed in religious tourism.	0.733			
	This religious tourism gave me positive feelings.	0.751			
	This religious tourism helped me feel acceptable.	0.766			
	This religious tourism improved the way people perceived me.	0.749			
	This religious tourism helped me to know more people.	0.724			
	This religious tourism increased my religious knowledge.	0.75			
	This religious tourism broadened my horizons.	0.763			
	This religious tourism helped to form good manners in my daily life.	0.744			
	Temples offered rich religious landscapes and architecture.	0.726			
	Temples offered vegetarian diets.	0.729			
	Temples provided the incense needed for worship.	0.77			
	Temples offered the opportunity to experience religious culture and atmosphere.	0.742			
	Temples offered multimedia events with religious themes.	0.76			
	Temples offered religious-themed programs.	0.726			
Awe (AW)	In the temple, I felt bored-excited.	0.928	0.945	0.961	0.859
	In the temple, I felt usual-unusual.	0.931			
	In the temple, I felt unexpected-expected.	0.926			
	In the temple, I felt arrogant-humbling.	0.923			
Subjective well-being (SW)	The experience of temple visiting made me feel delighted involuntarily.	0.916	0.953	0.964	0.842
	During my visit to temple, I felt like I was in high spirits and full of vigor.	0.923			
	I felt like my travel was satisfactory and festive.	0.91			
	This temple visiting experience helped me have warm, satisfying, and trustful relationships with others.	0.925			
	This temple visiting experience helped me become self-determining and independent.	0.913			
Religiosity (RE)	My religiosity is very important to me.	0.925	0.956	0.966	0.850
	My religion or faith is an important part of my identity.	0.922			
	If someone wants to understand who I am as a person, my religion or faith would be very important in knowing that.	0.921			
	I believe strongly in the teachings of my religion or faith.	0.919			
	I consider myself a religious person.	0.924			
Donation intention (DI)	I intend to donate resources (financial or in-kind) to the temple in the future.	0.908	0.940	0.957	0.848
	I plan to donate resources (financial or in-kind) to the temple in the near future.	0.931			
	I am willing to contribute by donating resources (financial or in-kind) to the temple.	0.916			
	I am committed to donating resources (financial or in-kind) to the temple.	0.928			

CA, Cronbach's Alpha; CR, Composite Reliability; AVE, Average Variance Extracted.

**Table 3:** Discriminant validity.

	**PV**	**AW**	**SW**	**RE**	**DI**
PV	0.749	*0.237*	*0.248*	*0.178*	*0.244*
AW	0.229	0.927	*0.450*	*0.158*	*0.435*
SW	0.239	0.428	0.917	*0.155*	*0.432*
RE	0.171	0.150	0.148	0.922	*0.365*
DI	0.235	0.412	0.411	0.346	0.921

PV, Perceived Value; AW, Awe; SW, Subjective Well-being; RE, Religiosity; DI, Donation Intention; Underline font, Square-root of the AVE; Italic font, Heterotrait-Monotrait Ratio.

### 4.4 Hypothesis testing

This study used 5,000 bootstrap samples to perform hypothesis testing. Table 4 presents the results of the PLS-SEM analysis. The results indicate that perceived value significantly and positively influences awe (β = 0.229, *p* < 0.001), subjective well-being (β = 0.149, *p* < 0.001), and donation intention (β = 0.073, *p* < 0.05), supporting H1, H2, and H3. Awe significantly and positively influences subjective well-being (β = 0.394, *p* < 0.001) and donation intention (β = 0.236, *p* < 0.001), supporting H4 and H5. Subjective well-being also significantly influences donation intention (β = 0.246, *p* < 0.001), supporting H6. These findings underscore the importance of tourists' overall perceptions at religious sites in fostering donation intentions.

**Table 4:** Structural model results.

**Hypothesis path**	**β**	**Std**.	***T* values**	***P* values**	**Results**
**Direct effect**					
H1: PV->AW	0.229^***^	0.043	5.265	0.000	Supported
H2: PV->SW	0.149^***^	0.040	3.774	0.000	Supported
H3: PV->DI	0.073^*^	0.036	2.050	0.040	Supported
H4: AW->SW	0.394^***^	0.044	8.960	0.000	Supported
H5: AW->DI	0.236^***^	0.044	5.386	0.000	Supported
H6: SW->DI	0.246^***^	0.045	5.467	0.000	Supported
**Mediating effect**					
H7: PV → AW → DI	0.055^***^ [0.029~0.087]	0.015	3.634	0.000	Supported
H8: PV → SW → DI	0.037^**^ [0.016~0.064]	0.012	3.019	0.003	Supported
**Moderating effect**					
H9: RE^*^AW → DI	0.136^**^	0.049	2.772	0.006	Supported
H10: RE^*^SW → DI	0.101^*^	0.047	2.156	0.031	Supported

^***^P < 0.001, ^**^P < 0.01, ^*^P < 0.05, [] indicates 95% confidence intervals.

The results in Table 4 further show that, through the mediating role of awe, perceived value significantly and positively influences donation intention (β = 0.055), with a 95% confidence interval of (.029, 0.087) (excluding 0), supporting H7. Similarly, subjective well-being also significantly and positively influences donation intention through perceived value (β = 0.037), with a 95% confidence interval of (0.016, 0.064) (excluding 0), supporting H8. These mediation effects highlight awe and subjective well-being as crucial mechanisms through which perceived value influences donation intentions.

Table 4 also presents the results of the moderation effects. Specifically, religiosity significantly and positively moderated the relationship between awe and donation intention (β = 0.136, *p* < 0.01), thus supporting H9. Additionally, religiosity positively moderated the relationship between subjective well-being and donation intention (β = 0.101, *p* < 0.05), further supporting H10. These moderation effects suggest that higher religiosity strengthens emotional responses, which in turn influence donation intentions, emphasizing the importance of religious beliefs in understanding prosocial behavior in religious tourism contexts.

## 5 Discussion

The current study offers an in-depth analytical framework and a comprehensive examination of how tourists' perceived value influences their intention to donate via awe and subjective well-being. Consistent with Lv and Xie (2017), our results demonstrate a positive relationship between perceived value and subjective well-being, emphasizing the beneficial effect of perceived value on personal well-being. This supports the core tenet of consumer value theory (Tanrikulu, 2021), which states that utility evaluations shape affective outcomes, but it also extends this theory by incorporating the spiritual and emotional dimensions unique to religious heritage tourism. Our study reaffirms that perceived value is a major driver of tourists' perceptions, attitudes, and behaviors (Chen and Chen, 2010; Eid, 2015; PandŽa Bajs, 2015) and highlights subjective well-being as an essential component of tourist experiences (Bagheri et al., 2024). By demonstrating that perceived value encompasses psychological and emotional experiences beyond material aspects, our findings challenge more utilitarian perspectives and support calls to broaden the conceptualization of value in tourism research.

Our findings suggest that perceived value has a significant impact on awe and tourists' donation intention. Awe arises when visitors encounter stimuli beyond their understanding, particularly in religious heritage tourism (Xu and Hu, 2024; Zhang and He, 2025). By empirically showing that awe mediates the link between perceived value and donation, this study corroborates the broaden-and-build theory of positive emotions (Fredrickson, 2001) and extends the SOR framework by underscoring the central role of emotions in driving prosocial behavior. It suggests that emotional investment grows in proportion to the perceived distinctiveness and cultural value of a destination. The strong effect of awe relative to subjective well-being on donation intention was somewhat unexpected; we anticipated both mediators to exert comparable influence, yet awe demonstrated a more pronounced impact. This implies that immediate, transcendent emotions may be more potent drivers of prosocial behavior than more diffuse assessments of life satisfaction, an insight that enriches the literature on emotion-driven prosociality (Song et al., 2023).

Perceived value, as an external stimulus, triggers tourists' emotional resonance and psychological identification, promoting donation behavior. Tourists' donation behaviors are primarily driven by their perceived value of the destination and their emotional engagement (Aji and Muslichah, 2023; Moran and Bagchi, 2019). Hence, higher perceived value enhances the likelihood of donation, especially in contexts of cultural heritage or environmental preservation.

It is worth highlighting the effect of awe on subjective well-being. Consistent with previous research by Liu et al. (2023) and Xu and Hu (2024), our study demonstrates that awe significantly enhances tourists' subjective well-being. Awe is a self-transcendent emotion triggered by encounters with vast or sacred stimuli (Keltner and Haidt, 2003), and positive psychology research shows that awe-related physiological and cognitive shifts are associated with increased optimism, a heightened sense of connection, and well-being (Monroy and Keltner, 2023). Awe also diminishes self-focus and promotes prosocial behaviors, such as cooperation, generosity, and volunteering, which are themselves linked to higher well-being (Monroy and Keltner, 2023). These findings suggest that when tourists experience awe at magnificent natural vistas or sacred sites, the emotion not only elicits immediate feelings of admiration and respect but also enhances their overall happiness and encourages altruistic actions. The observed increase in subjective well-being provides empirical support for the positive psychology claim that awe can improve mental health and foster selflessness (Monroy and Keltner, 2023).

A noteworthy contribution of this study is the identification of religiosity as a moderator. Religiosity was found to strengthen the positive effects of awe and subjective well-being on donation intention, suggesting that internalized beliefs amplify emotional responses and normative motivations. This finding confirms theories positing that religiosity enhances prosocial behavior (Domaradzki and Walkowiak, 2024) and supports evidence that individuals who view donation as a religiously good deed are more willing to contribute (Domaradzki and Walkowiak, 2024). It also refines prior debates by showing that religiosity acts not merely as a direct predictor but as a catalyst that conditions the influence of emotions on behavior. An unexpected nuance, however, is that religiosity's moderating effect was stronger on the awe–donation link than on the well-being–donation link. This suggests that spiritual beliefs may resonate more with transcendent emotions than with general life satisfaction, highlighting the importance of context-specific interpretations of religiosity. Future research could explore whether religiosity dampens prosocial responses when the cause is secular or misaligned with religious values, as some studies suggest negative effects in other domains.

Using the SOR theory, this research demonstrates how environmental stimuli enable tourists to transform emotions and cognition, ultimately affecting donation behavior. Religious heritage is both a cultural and spiritual symbol; it evokes respect and happiness through architecture and cultural connotations. These emotional experiences are not transient but facilitate a deeper psychological adjustment, converting tourists from passive observers into active supporters. Our findings confirm the applicability of SOR theory in heritage tourism, while challenging purely cognitive interpretations by highlighting emotion-driven pathways. They also point to the transformative potential of awe-induced self-transcendence in fostering social responsibility.

## 6 Conclusion

### 6.1 Theoretical contributions

The present study advances the theoretical landscape of religious tourism and donor behavior in several novel ways. First, this study introduces donation intention as a behavioral outcome within the SOR framework. Whereas most SOR applications in tourism examine purchase or visit intentions (Hew et al., 2018; Zhu et al., 2024), we explicitly focus on donation behavior. By demonstrating the impact of tourists' perceived value on their donation intentions in the context of religious tourism, the study broadens the theoretical lens from consumerism to altruism, addressing a gap in existing research. Donation behavior in this context reflects both participation in religious rituals (Toffin, 2015) and emotional connection to destinations (Hu et al., 2024), providing a more holistic understanding of visitor engagement.

Second, the study's integrated examination of awe and subjective well-being as dual mediators is conceptually innovative. Whereas previous research has often treated awe as an isolated emotional reaction (Lu et al., 2017; Wang et al., 2021), our findings demonstrate that awe interacts with perceived value to simultaneously enhance subjective well-being and trigger prosocial motives. This dual-pathway insight expands the applicability of SOR theory by highlighting how emotional and cognitive processes jointly explain donation behavior.

Third, we foreground religiosity as a key moderating factor that amplifies the influence of awe and subjective well-being on donation intentions. Prior studies have rarely positioned religiosity as a moderator within a tourism framework; by doing so, we reveal that spiritual commitment intensifies the moral and emotional resonance of awe, thereby catalyzing prosocial behavior. In accordance with previous studies by Abror et al. (2019), Bhandari et al. (2024), and Rahman et al. (2022), this finding deepens theoretical understanding of how personal belief systems shape the translation of emotional experiences into behavioral responses.

### 6.2 Practical implications

The study also offers actionable insights for stakeholders in religious heritage tourism. For site managers, the findings underscore the importance of designing visitor experiences that evoke awe and meaning, which not merely to enhance satisfaction but to inspire charitable support. Investing in high-quality interpretive services, immersive storytelling, and reflective rituals can amplify perceived value and emotional engagement, thereby boosting donation intent. Managers should recognize that fostering awe and well-being is not solely a cultural imperative but also a strategic pathway to sustainable funding.

For destination marketing organizations, the moderation effect of religiosity suggests tailoring communications to visitors' spiritual orientations. Highly religious tourists may respond positively to messages emphasizing spiritual participation and moral duty, whereas less religious visitors may be more motivated by narratives about cultural heritage and social responsibility. Segmentation strategies based on religiosity can thus optimize both visitor experience and fundraising outcomes.

## 7 Limitations and future research

This study has several limitations. First, data were collected solely from tourists visiting the A-Ma Temple in Macau, which limits the generalizability of the findings to other religious or cultural settings. Second, the cross-sectional design restricts the ability to draw strong causal inferences. Third, the research model did not include any control variables and did not account for other potentially influential factors such as prior religious tourism experience, nationality, or cultural background. Fourth, all measures relied on self-report questionnaires, which may be subject to social desirability bias, particularly regarding religiosity and donation intentions.

Future studies should consider collecting data from a wider range of religious heritage sites and diverse cultural contexts to improve generalizability. Longitudinal or experimental designs are recommended to better establish causality. Researchers are also encouraged to include and compare additional psychological variables (such as self-transcendence, altruism, or emotional attachment) and cultural factors (such as cultural orientation or collectivism/individualism) to further clarify the mechanisms influencing donation intention. Finally, the use of mixed methods or behavioral measures could help reduce bias associated with self-report data and provide richer insights into tourist donation behavior.

## Data Availability

The original contributions presented in the study are included in the article/Supplementary material, further inquiries can be directed to the corresponding author.
