# Supplementary material for: Perceived value and tourist donation intentions at religious heritage sites: the mediating roles of awe and subjective well-being
Source: Front Psychol. 2025 Sep 12;16:1655120. doi: 10.3389/fpsyg.2025.1655120 (PMC12463621; doi:10.3389/fpsyg.2025.1655120)
Supplement: Supplementary file 1 [file Table_1.DOCX]

Supplementary Material

# Supplementary appendix

## 3.1 Translation and Cross‑Cultural Adaptation of Measurement Instruments

This report documents the translation and validation process used to adapt the measurement instruments for our study on religious heritage tourism. Following best practice guidelines for cross‑cultural adaptation (Thammaiah et al., 2016), we adopted a multi‑step procedure that emphasized linguistic precision, cultural appropriateness and psychometric robustness. Below, each step is outlined in chronological order to provide a transparent account of how the original English scales were transformed into a reliable and valid Chinese version.

**Step 1 – Adaptation and creation of Chinese version v1**

We began by adapting the original English items to the research context of visiting a religious temple. The aim at this stage was to produce an initial Chinese version (v1) that was faithful to the constructs while reflecting the cultural nuances of temple visits. Key changes included:

Perceived value: Phrases referring to generic “resorts” were replaced with “寺庙” (“temple”), and service quality items were tailored to highlight interpersonal warmth appropriate for sacred settings. For instance, the original item “The environment of the resort was good” was adapted to “寺庙的环境很好” (“The environment of the temple was good”). Similarly, “The resort has well-developed infrastructure” was revised to “寺庙的基础设施完善” (“The temple had well-developed infrastructure”), emphasizing the adaptability to religious sites.

Awe: Each semantic differential pair (e.g., “boring–exciting”) was contextualized by adding the stem “在寺庙中，我感到…” (“In the temple, I felt…”). This ensured respondents anchored their feelings to the temple experience. For example, “无聊‑兴奋” (“boring‑excited”) became “在寺庙中，我感到无聊‑兴奋” (“In the temple, I felt bored-excited”).

Subjective well‑being and religiosity: Original statements already referred to temple visits, so translation was direct. For example, translate “The experience of temple visiting made me feel delighted involuntarily” as “参观寺庙的经历让我不由自主地感到愉悦.”; translate “I consider myself a religious person.” as “我认为自己是一个虔诚的人.”

Donation intention: Donation intention items were modified from “food donation” to “捐赠资源（资金或实物）,” indicating monetary or in‑kind contributions to the temple. For example, “I intend to donate food in the future” was changed to “我打算将来向寺庙捐赠资源（资金或实物）”(“I intend to donate resources (financial or in-kind) to the temple in the future”).

This adaptation stage generated the first Chinese version (v1), ready for back‑translation and further refinement.

**Step 2 – Back‑translation cycle and reconciliation**

Cross‑cultural guidelines recommend a rigorous back‑translation process to ensure semantic equivalence (Thammaiah et al., 2016). In our procedure, two bilingual tourism professors participated:

Forward (English) translation from Chinese v1: Translator A, a native Chinese speaker and tourism professor from Macao City University, translated the Chinese v1 into English. This “forward” translation produced an English version that reflected how the Chinese items would be expressed in English.

Backward (Chinese) translation: Translator B, a native English speaker and tourism professor from Macao University of Science and Technology who had never seen the original English scales, translated Translator A’s English version back into Chinese, yielding a “Chinese back‑translation” (v2). Because Translator B lacked prior exposure to the original items, any shifts in meaning introduced during adaptation were likely to surface in v2.

Comparison and revision: The research team compared v1 and v2 line by line. Whenever meanings diverged, the team considered whether the divergence resulted from mistranslation, cultural adjustment or conceptual drift. Where necessary, v1 was adjusted and the cycle repeated until the back‑translated version no longer differed materially from the adapted v1.

Examples of discrepancies and adjustments:

Service quality: The v1 item “寺庙工作人员提供优质服务” (“The temple staff provided high‑quality service”) was back‑translated as “The temple staff provided high‑quality service.” Although correct, the nuance of patience and guidance, important in a sacred context, was lost. To recover this nuance, we revised the Chinese item to “寺庙工作人员耐心回答了我的问题,” explicitly mentioning patience. This rewording produced the desired back translation “The temple staff patiently answered my questions.”

Transportation item: The v1 phrasing “寺庙的交通便利且经济实惠” (“The transportation of the temple is convenient and affordable”) was back‑translated as “The transportation around the temple is convenient and cheap,” suggesting mobility within the temple grounds. We clarified the intended meaning by specifying “前往寺庙的交通便利且经济实惠” (“Transportation to the temple was convenient and affordable.”), which back‑translated accurately.

Awe semantic pair: The pair “傲慢‑谦卑” (arrogant–humble) in v1 yielded the back translation “arrogant–submissive,” implying obedience rather than humility. We replaced “谦卑” with “谦逊,” capturing humility without connotations of subservience, thus aligning the semantic differential more closely with the original concept of “arrogant–humbling.”

Subjective well‑being: In v1, the relationship item used “温暖、令人满足和信任的关系” (“warm, satisfying and trusting relationships”), but the back translation omitted “trust.” To ensure that trust was explicitly conveyed, we refined the item to “温暖、令人满足且令人信赖的关系,” which back‑translated as “warm, satisfying and trustful relationships.”

The above examples illustrate how the back‑translation process exposed subtle semantic mismatches and allowed us to refine v1. This iterative procedure—translation, back translation, comparison and revision—was repeated until the research team concluded that v1 and v2 conveyed identical meanings.

**Step 3 – Expert review and bilingual focus group**

Once the back‑translation cycle yielded a stable Chinese version, we sought additional validation from experts and prospective respondents. Two tourism scholars familiar with measurement development but independent of the translation team reviewed each item for conceptual equivalence, clarity and cultural relevance, in keeping with guidelines that recommend expert committee review (Thammaiah et al., 2016). They rated each item on relevance and clarity using a four‑point scale. The item‑level content validity index (I‑CVI) ranged from 0.83 to 1.00, and the scale‑level average (S‑CVI/Ave) was 0.95, indicating high content validity. Feedback focused on ensuring colloquial readability (e.g., replacing formal expressions with everyday language) and reinforcing the spiritual connotations of certain phrases.

To further examine cultural intelligibility, we conducted a focus discussion with bilingual temple visitors. Participants read each item and commented on its clarity and appropriateness. Their observations helped fine‑tune a few items (e.g., clarifying that “庆祝氛围” signified a general sense of joy rather than association with a particular festival). No substantive changes were required, reinforcing that the items were well understood.

**Step 4 – Pretest and psychometric evaluation**

The refined questionnaire underwent a pilot test to assess reliability and validity. Seventy‑four visitors to the A‑Ma Temple completed the survey. Respondents were invited to note any confusing or culturally inappropriate items; none were reported. Based on the pilot data, Cronbach’s α values exceeded 0.80 for all constructs (perceived value: 0.979; awe: 0.945; subjective well‑being: 0.954; religiosity: 0.960; donation intention: 0.948). Composite reliability values exceeded the recommended 0.70 threshold, and average variance extracted (AVE) values were greater than 0.50 (see Table S1). In addition, the square root of the AVE for each construct was greater than the correlation between it and the other constructs. All of the heterogeneous-to-monomorphic (HTMT) ratios were below 0.85 (see Table S2). These findings confirm that the translated scales achieved acceptable reliability and validity. The pretest thus fulfils the guidelines’ recommendation to conduct a pre‑final test to verify comprehensibility and psychometric properties (Thammaiah et al., 2016).

**Conclusion**

By following a systematic adaptation, translation and validation process, we ensured that the measurement instruments used in this study are culturally appropriate, semantically equivalent to the originals and psychometrically sound. Starting with adaptation of the English items to produce a Chinese v1, we employed a two‑stage back‑translation cycle involving bilingual tourism professors who were native speakers of Chinese and English, respectively. The iterative reconciliation of v1 and v2 resolved subtle semantic differences—such as clarifying service quality nuances, distinguishing transportation to versus within the temple, refining semantic differential pairs and emphasising trust in interpersonal relations. Subsequent expert review and bilingual focus‑group feedback further verified conceptual alignment and cultural intelligibility, while a pilot test with 74 participants demonstrated high reliability and validity. This rigorous approach provides confidence that the final Chinese questionnaire accurately measures perceived value, awe, subjective well‑being, religiosity and donation intention in the context of religious heritage tourism.

**Table S1.** Respondent demographics (Pre-testing).

| Variable | Category | Frequency (N=74) | Percent% (N=74) |
| --- | --- | --- | --- |
| Gender | Male | 30 | 40.5 |
|  | Female | 44 | 59.4 |
| Age | 18-25 years old | 16 | 21.6 |
|  | 26-35 years old | 18 | 24.3 |
|  | 36-45 years old | 17 | 22.9 |
|  | 46-55 years old | 10 | 13.5 |
|  | 56 years old and above | 13 | 17.5 |
| Education | Junior high school and below | 8 | 10.8 |
|  | High School/Secondary School | 16 | 21.6 |
|  | University/Junior college | 41 | 55.4 |
|  | Postgraduate degree | 9 | 12.1 |
| Income (RMB) | Under 3000 | 14 | 18.9 |
|  | 3001-5000 | 25 | 33.7 |
|  | 5001-10000 | 20 | 27.0 |
|  | Above 10001 | 15 | 20.2 |
| Career | Student | 10 | 13.5 |
|  | Company employees | 22 | 29.7 |
|  | Self-employed | 14 | 18.9 |
|  | Institutions/civil servants | 11 | 14.8 |
|  | Service industry personnel | 8 | 10.8 |
|  | Workers | 1 | 1.3 |
|  | Others | 8 | 10.8 |

**Table S2.** Reliability and validity analysis of scale (Pre-testing).

| Construct | Item | Factor Loading | CA | CR | AVE |
| --- | --- | --- | --- | --- | --- |
| Perceived Value  (PV) | The environment of the temple was good. | 0.781 | 0.979 | 0.980 | 0.703 |
|  | The temple had well-developed infrastructure. | 0.772 |  |  |  |
|  | The temple staff patiently answered my questions. | 0.777 |  |  |  |
|  | The price of the city where the temple was located was reasonable. | 0.843 |  |  |  |
|  | Transportation to the temple was convenient and affordable. | 0.843 |  |  |  |
|  | Spending at temples (meals, cultural creations, etc.) was reasonable. | 0.840 |  |  |  |
|  | I was comfortable with this religious tourism. | 0.830 |  |  |  |
|  | I felt relaxed in religious tourism. | 0.788 |  |  |  |
|  | This religious tourism gave me positive feelings. | 0.811 |  |  |  |
|  | This religious tourism helped me feel acceptable. | 0.828 |  |  |  |
|  | This religious tourism improved the way people perceived me. | 0.869 |  |  |  |
|  | This religious tourism helped me to know more people. | 0.817 |  |  |  |
|  | This religious tourism increased my religious knowledge. | 0.894 |  |  |  |
|  | This religious tourism broadened my horizons. | 0.859 |  |  |  |
|  | This religious tourism helped to form good manners in my daily life. | 0.879 |  |  |  |
|  | Temples offered rich religious landscapes and architecture. | 0.891 |  |  |  |
|  | Temples offered vegetarian diets. | 0.890 |  |  |  |
|  | Temples provided the incense needed for worship. | 0.811 |  |  |  |
|  | Temples offered the opportunity to experience religious culture and atmosphere. | 0.884 |  |  |  |
|  | Temples offered multimedia events with religious themes. | 0.834 |  |  |  |
|  | Temples offered religious-themed programs. | 0.850 |  |  |  |
| Awe  (AW) | In the temple, I felt bored-excited. | 0.924 | 0.945 | 0.960 | 0.858 |
|  | In the temple, I felt usual-unusual. | 0.943 |  |  |  |
|  | In the temple, I felt unexpected-expected. | 0.928 |  |  |  |
|  | In the temple, I felt arrogant-humbling. | 0.909 |  |  |  |
| Subjective Well-being  (SW) | The experience of temple visiting made me feel delighted involuntarily. | 0.931 | 0.954 | 0.964 | 0.843 |
|  | During my visit to temple, I felt like I was in high spirits and full of vigor. | 0.923 |  |  |  |
|  | I felt like my travel was satisfactory and festive. | 0.905 |  |  |  |
|  | This temple visiting experience helped me have warm, satisfying, and trustful relationships with others. | 0.918 |  |  |  |
|  | This temple visiting experience helped me become self-determining and independent. | 0.915 |  |  |  |
| Religiosity  (RE) | My religiosity is very important to me. | 0.916 | 0.960 | 0.969 | 0.861 |
|  | My religion or faith is an important part of my identity. | 0.931 |  |  |  |
|  | If someone wants to understand who I am as a person, my religion or faith would be very important in knowing that. | 0.944 |  |  |  |
|  | I believe strongly in the teachings of my religion or faith. | 0.917 |  |  |  |
|  | I consider myself a religious person. | 0.931 |  |  |  |
| Donation Intention  (DI) | I intend to donate resources (financial or in-kind) to the temple in the future. | 0.919 | 0.948 | 0.962 | 0.865 |
|  | I plan to donate resources (financial or in-kind) to the temple in the near future. | 0.932 |  |  |  |
|  | I am willing to contribute by donating resources (financial or in-kind) to the temple. | 0.937 |  |  |  |
|  | I am committed to donating resources (financial or in-kind) to the temple. | 0.932 |  |  |  |

Note: CA = Cronbach’s Alpha, CR = Composite Reliability, AVE = Average Variance Extracted.

**Table S3.** Discriminant validity (Pre-testing).

|  | PV | AW | SW | RE | DI |
| --- | --- | --- | --- | --- | --- |
| PV | 0.838 | *0.202* | *0.155* | *0.167* | *0.346* |
| AW | 0.206 | 0.926 | *0.628* | *0.152* | *0.339* |
| SW | 0.161 | 0.602 | 0.918 | *0.063* | *0.413* |
| RE | 0.166 | 0.133 | 0.006 | 0.928 | *0.569* |
| DI | 0.347 | 0.321 | 0.393 | 0.544 | 0.930 |

Note: PV = Perceived Value; AW = Awe; SW = Subjective Well-being; RE = Religiosity; DI = Donation Intention; Underline font = Square-root of the AVE; Italic font = Heterotrait-Monotrait

**References**

Thammaiah, S., Manchaiah, V., Easwar, V., & Krishna, R. (2016). Translation and adaptation of five English language self-report health measures to South Indian Kannada language. *Audiology research*, *6*(1), 153.
